# Aircraft Noise Reduction Strategies and Analysis of the Effects

**DOI:** 10.3390/ijerph20021352

**Published:** 2023-01-11

**Authors:** Jinlong Xie, Lei Zhu, Hsiao Mun Lee

**Affiliations:** School of Mechanical and Electrical Engineering, Guangzhou University, 230 Wai Huan Xi Road, Guangzhou 510006, China

**Keywords:** noise map, Guangzhou Baiyun International Airport, aircraft noise

## Abstract

In this study, six aircraft noise reduction strategies including the optimization of aircraft type, regulation of night flight number, optimization of flight procedure, modification of operating runway, land use planning and installation of sound insulation windows were proposed to alleviate the harmful impact of aircraft noise on the local area and population near Guangzhou Baiyun International Airport (BIA) in China. The effects of all proposed strategies except for land use planning and sound insulation windows were simulated and analyzed using CadnaA software. The results indicate that these noise reduction strategies have their own advantages and each of them can serve as an effective noise reduction measure for different applications. For instance, the replacement of noisy aircraft with low-noise aircraft can simultaneously reduce the area and population exposed to a high noise level, while the optimization of flight procedure can only reduce the population exposed under relatively low noise levels ($70\;\leq L_{\mathrm{WECPN}}$ ≤ 75 dB). Nevertheless, the modification of operating runway is more effective in reducing the population suffering under high noise levels ($L_{\mathrm{WECPN}}$ > 85 dB). Among these strategies, reducing the number of night flights is found to be most effective in reducing the overall noise-exposed area and population. Additionally, with the assistance of noise mapping, proper land use planning was suggested according to national standards, and the installation of sound insulation windows with different sound reduction grades can be determined for different areas impacted by the aircraft noise of BIA. It is believed that the results of this study can be applied as a reference in selecting suitable noise reduction strategies to improve the acoustic environment of a specific airport.

## 1. Introduction

According to the latest report of the International Air Transport Association [1], global air passenger traffic is expected to exceed the pre-pandemic level in 2024. A forecast report by Airbus [2] predicted that global air passenger traffic will continue to grow at an annual growth rate of 3.6% from 2019 to 2041. Therefore, the global air transport industry will continue to grow rapidly in the future, which will further aggravate global aircraft noise pollution. As we all know, aircraft noise will affect the health of residents around the airport. Existing research showed that aircraft noise will damage people’s hearing [3,4], increase level of worries [5,6], interfere with people’s sleep [7,8] and affect their mental health [9]. In addition, aircraft noise will increase the probability of attention deficit hyperactivity disorder among children [10] and even increase the risk of cardiovascular diseases such as hypertension and coronary heart disease [11,12].

Nowadays, countries all over the world have promulgated aircraft noise regulations to control aircraft noise pollution, which is forcing airports to adopt various noise reduction measures. For example, the European Union environmental noise regulation 2002/49/EC [13] requires Member States to update the noise map every five years to assess the pollution level of aircraft noise. Up to 2009, at least 615 airports around the world had conducted noise reduction measures; most of them were Western countries [14]. Some airports around the world use fines or impose noise taxes in order to encourage airlines to adopt quieter aircraft [15]. Sound insulation measures for surrounding buildings taken by several airports in Spain were able to reduce the impact of aircraft noise on communities [16]. The findings of Postorino et al. [17] and Prats et al. [18] showed that optimizing the takeoff track of aircraft could effectively reduce the impact of aircraft noise on the population in noise-sensitive areas. The investigation by Vogiatzis [19] and Licitra et al. [20] showed that properly extending the runway could reduce the noise-exposed population. In addition, reasonable land use planning can effectively control aircraft noise pollution and is advantageous to the sustainable development of the airport [19,21].

From the abovementioned studies, it can be seen that the majority of these studies are conducted in Western countries. Relevant studies in China are still scarce. Lei et al. [22] evaluated the aircraft noise impacts of the third runway in Pudong International Airport. Investigations including the influences of runway location, optimized land use, selection of low noise aircrafts and Fly Quit Program were conducted. It was suggested that the restriction of high-noise aircrafts and the applications of a quiet aircraft operation program should be adopted. The same conclusions were also reported by Zhang et al. [23]. In their study, the installation of soundproof windows was suggested to mitigate aircraft noise in residential areas near Beijing Capital Airport. It was found that the overall sound insulation can reach as high as 10 dBA. Chen et al. [24] reported that for existing airports, expansion of existing ones and construction of new airports, the most effective strategy to reduce the impact of aircraft noise on local residential buildings was to design a plan for proper land use.

Up to now, studies on applying different noise reduction strategies for aircraft noise in airports have been numerous. However, systematic investigation of different noise reduction strategies for a jumbo international aviation hub is still scarce. In the present study, six noise reduction strategies including the optimization of aircraft type, regulation of number of night flights, flight procedure, modification of operating runway, land use planning and the installation of sound insulation windows were proposed to reduce the impacts of aircraft noise on the area and population near Guangzhou Baiyun International Airport (BIA) which is the international aviation hub in South China [25]. In 2020, the air passenger traffic of BIA exceeded that of Atlanta Airport, becoming the busiest airport in the world [26]. In our previous study [27], it was found that the acoustic environment quality of BIA was marginally acceptable because about 22.22% and 25.46% of the total population around BIA were exposed to the weighted equivalent continuous perceived noise level ($L_{\mathrm{WECPN}}$) > 70 dB. This is the other motivation of the current study, to evaluate an effective strategy to improve the acoustic conditions of BIA. Furthermore, the results of this study can also provide useful noise reduction guidelines for other jumbo airports.

## 2. Methodologies and Discussion of the Results

### 2.1. Optimization of the Aircraft Type

**Figure 1:**
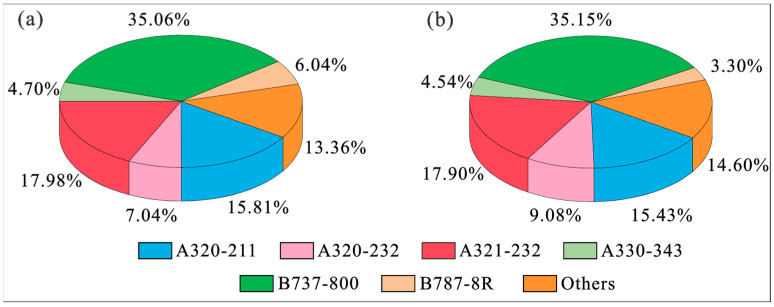
Proportions of the aircraft types that take off and land at BIA during (**a**) summer and (**b**) winter [28].

The first strategy proposed to reduce aircraft noise is to optimize aircraft types in the takeoff and landing stages. In the present study, the flight data of BIA during the summer and winter from 14th to 20th December 2020 and from 25th to 31st March 2021 were respectively collected to analyze the aircraft types that were operating in the airport. As shown in Figure 1, the characteristics of the aircraft types during takeoff and landing at BIA are presented. For all these aircrafts, their respective models recorded in this study are tabulated in Table A1 in the Appendix A. It can be seen that the differences of the aircraft types during summer and winter are very similar for BIA. The main aircraft models are B737-800, A321-232, A320-211, A320-232, A330-343 and B787-8R. These aircraft types account for 86.64% and 85.40% of the total aircrafts during the summer and winter, respectively. Among them, B737-800 is the most popular aircraft type found in BIA (about 35%), and hence is supposed to exert a significant impact on the overall acoustic environment.

**Figure 2:**
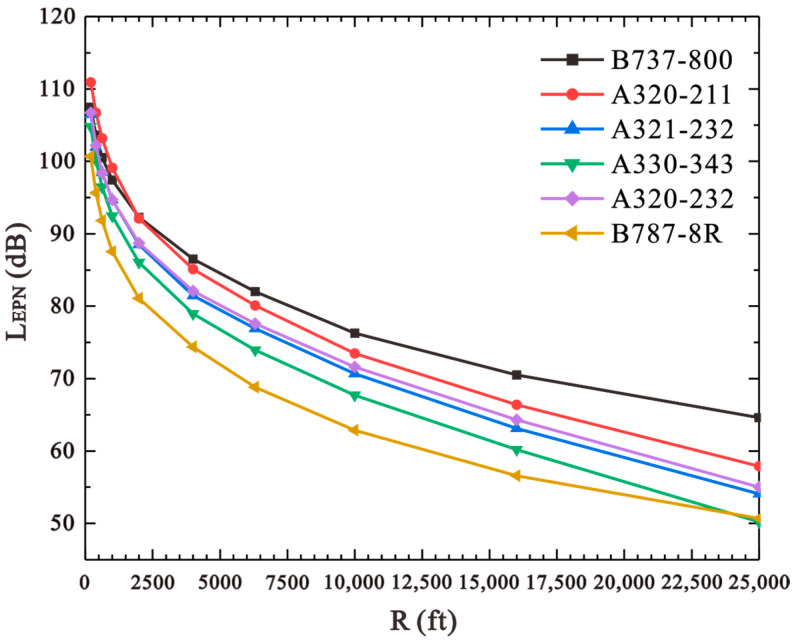
NPD curve of the main aircraft types at BIA (thrust = 19,000 lb).

In order to assess the noise level of different aircraft types, the Noise–Power–Distance (NPD) measurement of the main aircraft types at BIA was analyzed using the data from the aircraft noise and performance (ANP) database (Eurocontrol Experimental Centre (EEC) [29]), as shown in Figure 2. It is obvious that at the same distance between the measurement point and the aircraft track (R), the effective perceived noise level ($L_{\mathrm{EPN}}$) of B737-800 is at least 5 dB higher than that of A320-232, and 10 dB higher than that of B787-8R. It is suggested that replacing B737-800 (which is the main type in BIA) with low-noise aircraft types such as A320-232 or B787-8R could be a potential solution to improve the overall acoustic conditions of the airport. In order to match the passenger capacity and unit price of B737-800, the relative data of some popular aircraft types were sourced and summarized in Table 1. By combining Figure 2 and Table 1, A320-232 was finally selected as the candidate to replace B737-800 in the following noise level assessment.

With all the collected information, CadnaA software was then employed to construct noise maps before and after replacement with low-noise aircrafts. Details of the numerical methods can be found in the previous study [27].

**Figure 3:**
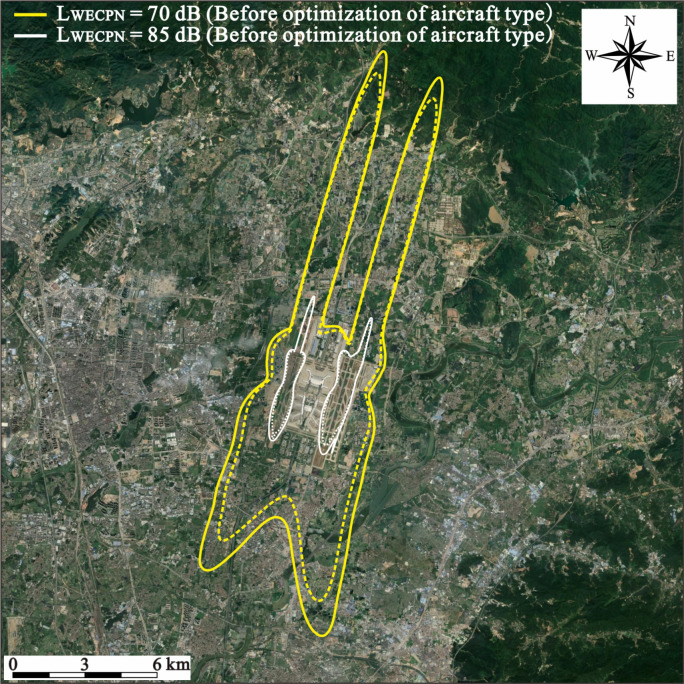
Noise contours before and after using the low-noise aircraft type during summer.

**Figure 4:**
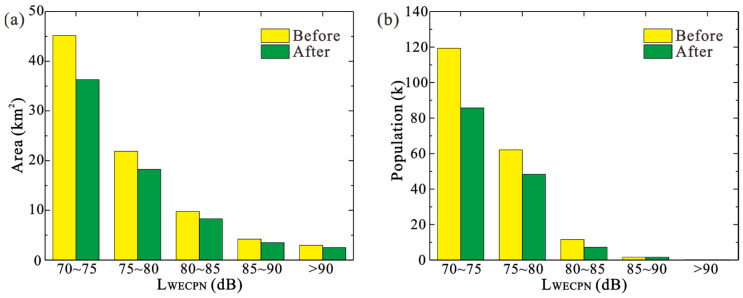
Noise reduction effects before and after using the low-noise aircraft type during summer: (**a**) area; (**b**) population.

Since the aircraft types in the summer and winter at BIA are quite similar, as shown in Figure 1, only the summer scenario was investigated. As shown in Table 2, and Figure 3 and Figure 4, the noise reduction effects due to the replacement of aircraft types during the summer are presented. As indicated, the overall area and population that are exposed to different noise levels are reduced significantly, implying that the strategy of using low-noise aircraft types can effectively reduce the noise level of BIA. In particular, the total exposed area and population with $L_{\mathrm{WECPN}}$ > 70 dB are decreased by 17.90% and 26.61%, respectively. Nevertheless, for residents who are living close to the runway of the airport and hence usually suffer from the high noise levels, i.e., $L_{\mathrm{WECPN}}$ > 85 dB, replacing the aircraft type will not help much. Overall, it is still worthwhile for BIA to formulate relevant measures to encourage airlines to adopt low-noise aircrafts in order to improve its acoustic conditions.

### 2.2. Regulation of Night Flight Numbers

The other practical scheme to alleviate the harmful effects of airport noise on nearby residents is to regulate the number of night flights [14,15]. In the present study, the influence of night flights on the overall $L_{\mathrm{WECPN}}$ of BIA was investigated. The assessment of $L_{\mathrm{WECPN}}$ was according to the national standard GB9661-88 [31], and the equation to calculate $L_{\mathrm{WECPN}}$ is shown in the equation below.

(1) $$L_{\mathrm{WECPN}}={\overset{ˉ}{L}}_{\mathrm{EPN}\;}+10\mathrm{lg}\left(N_{1\;}{+3\;N}_{2}{+10\;N}_{3}\right)-39.4$$

where $L_{\mathrm{EPN}}$ is the energy average of effective perceived noise level, N_1_, N_2_ and N_3_ are the number of aircraft during the day (7:00∼19:00), evening (19:00∼22:00) and night (22:00∼7:00), respectively.

**Figure 5:**
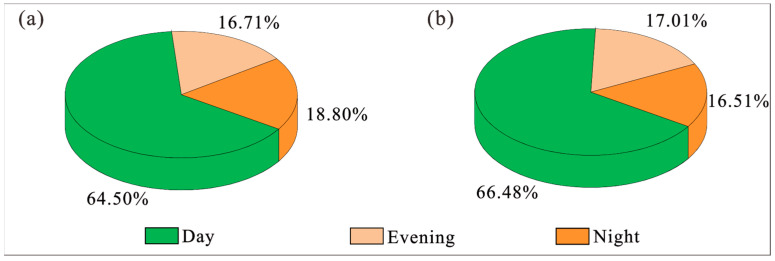
Proportions of the number of day, evening and night flights at BIA during (**a**) summer and (**b**) winter [28].

To conduct the analysis, the daily average number of flights at BIA was collected from the airport control center. The percentages of the number of flights in the day, evening and night are summarized in Figure 5. It is noted that, similar to the aircraft types in summer and winter, the percentages of the flight numbers in different time periods are similar in the summer and winter as well. In particular, night flights during summer and winter are 227 and 200, which correspond to 18.80% and 16.51% of the total flights, respectively. From the coefficients of Equation (1), it can be understood that the contribution of flight events at night on $L_{\mathrm{WECPN}}$ is much greater than that during the day and evening. Hence, although the percentage of flights at night is significantly lower than that in the daytime, the impact of flights at night is suggested to be much higher than those of daytime flights on the overall noise level of the airport. To investigate the influence of night flights, this study has evaluated the impacted area and population according to two scenarios as shown below. Although these two scenarios may not be feasible in practice immediately, the results can still be used as a reference for flight planning in future for similarly large airports.

1. Practice 1: transfer 50% of night flights to day and evening;
2. Practice 2: transfer all night flights to day and evening (implement a curfew).

**Figure 6:**
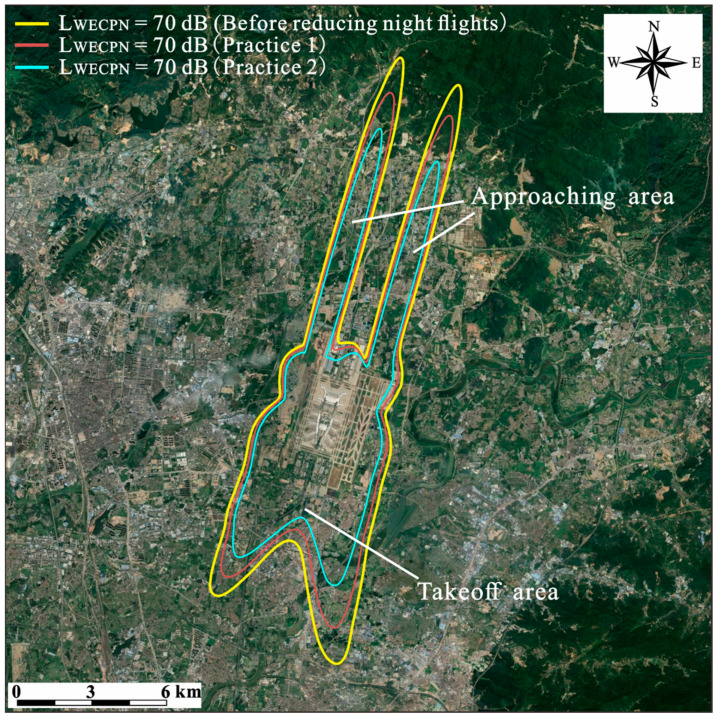
Noise contours before and after the reduction in night flights in two practices during summer.

**Figure 7:**
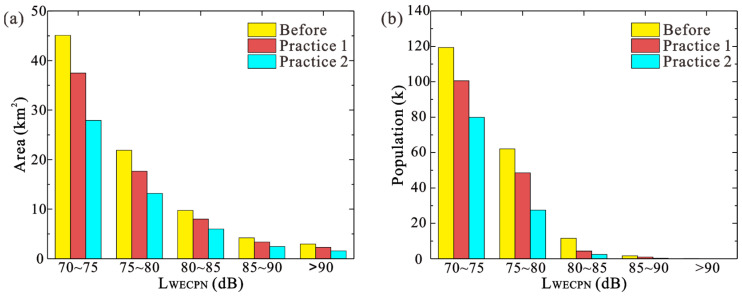
Noise reduction effects before and after the reduction in night flights in two stages during summer. (**a**) area (**b**) population.

Similar to Section 2.1, the noise maps of BIA under different numbers of night flights were simulated using CadnaA software. The results are demonstrated in Table 3, and Figure 6 and Figure 7. Again, only cases during summer were investigated due to the similar daily average numbers of flights in summer and winter. From Table 3, it can be found that with transferring half of night flights (Practice 1), the exposed area and population under $L_{\mathrm{WECPN}}$ > 70 dB can be decreased by 17.97% and 20.54%, respectively. With increasing $L_{\mathrm{WECPN}}$, the effects of reducing night flights become more significant, i.e., under $L_{\mathrm{WECPN}}$ > 90 dB, the exposed population can be decreased by 77.42%, which is quite pronounced. By further transferring night flights to day and evening, i.e., in Practice 2, the exposed area and population can be further decreased as expected. The results show that in Practice 2 under $L_{\mathrm{WECPN}}$ > 70 dB, the exposed area and population can be reduced by 39.03% and 43.43%, respectively, which are about twice the effect of Practice 1. Particularly, for $L_{\mathrm{WECPN}}$ > 90 dB, there would be no people suffering under such a high noise level under Practice 2. 

Figure 6 shows the space areas before and after the implementation of Practice 1 and 2 under $L_{\mathrm{WECPN}}$ > 70 dB. As can be seen, the main areas influenced by regulating night flight numbers are largely in the takeoff and approaching areas, while in the area that is close to the airport, that influence becomes negligible. It is suggested that the planning of flights should be considered together with land use planning in order to minimize the impact of aircraft noise. Figure 7 further illustrates the comparisons of the impacted area and population under different noise levels with/without the regulation of night flights. It is obvious that both the impacted area and population decrease with the decreasing of night flights, particularly for populations suffering under a higher noise level.

### 2.3. Optimization of the Flight Procedure

**Figure 8:**
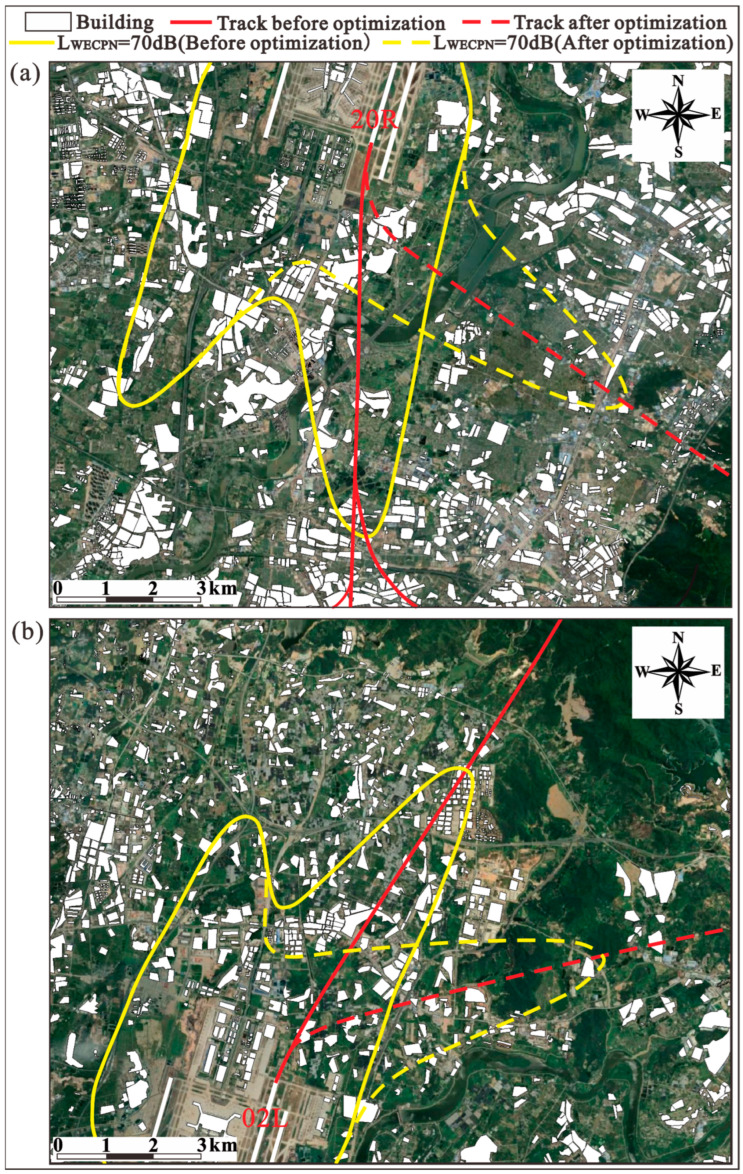
Noise contours before and after optimization of flight procedure during (**a**) summer and (**b**) winter.

In general, people will be seriously affected by aircraft noise if an aircraft just flies over them. Hence, for an airport where thousands of flights take off or land daily, the nearby residents are more easily exposed to the aircraft noise. Logically, the total population that is affected by aircraft noise can be reduced if the aircraft avoids passing through the densely populated area near an airport. For BIA specifically, the buildings below the takeoff track of runway 02L/20R are sparsely distributed (refer to Section 2.1 in Lee et al. [27] for the details of this runway). Thus, the population affected by the aircraft noise of BIA can be reduced by optimizing the takeoff track of runway 02L/20R. As shown in Figure 8, optimized takeoff tracks in summer and winter were proposed according to current geographical conditions near BIA. In particular, the red solid line is the existing takeoff track, while the red dash line is the optimized takeoff track.

The modified flight procedure according to geographical conditions is as follows.

3. **Summer:** Change the flight procedures of T-20R-A, T-20R-B and T-20R-C (refer to Section 2.3 in Lee et al. [27] for the details of flight procedure) of runway 20R to “after taking off from runway 20R, turn left at 71° (turning radius = 2.0 km), then fly straightly......”, as shown in Figure 8a.
4. **Winter:** Change the flight procedures of T-02L-A, T-02L-B and T-02L-C of runway 02L to “after taking off from runway 02L, turn right at 61° (turning radius = 2.0 km), then fly straightly......”, as shown in Figure 8b.

With the information on existing and optimized takeoff tracks, noise reduction effects due to modified flight procedure in summer and winter were evaluated using CadnaA software. In Figure 8, the yellow solid line covers the area under $L_{\mathrm{WECPN}}$ > 70 dB for the existing takeoff track, and the yellow dashed line covers the area under $L_{\mathrm{WECPN}}$ > 70 dB for the optimized takeoff track. In Table 4, the differences of the impacted areas and populations under the existing and optimized tracks are summarized. It can be seen that the areas under different noise levels remain almost the same before and after the modified flight procedure. This is expected, since the overall flights and aircraft types are the same before and after the change in flight procedure. Nevertheless, the exposed populations in the noise-affected areas in summer and winter under $L_{\mathrm{WECPN}}$ > 70 dB can be reduced by 18.50% and 10.94%, respectively. This implies that a simple optimization of flight procedure can easily reduce the impact of aircraft noise on surrounding residents to a certain extent, which is quite promising. From Table 4, it can be also found that for the residents suffering under a higher $L_{\mathrm{WECPN}}$ (i.e., $L_{\mathrm{WECPN}}$ > 80 dB), the effects of the modified takeoff track become negligible. This is because, for populations who suffer under a higher $L_{\mathrm{WECPN}}$, they are usually residing in areas close to the airport. Due to the nature of flight procedure, it is difficult to avoid aircraft noise in these areas by changing the path of a takeoff track.

### 2.4. Modification of the Operating Runway

**Figure 9:**
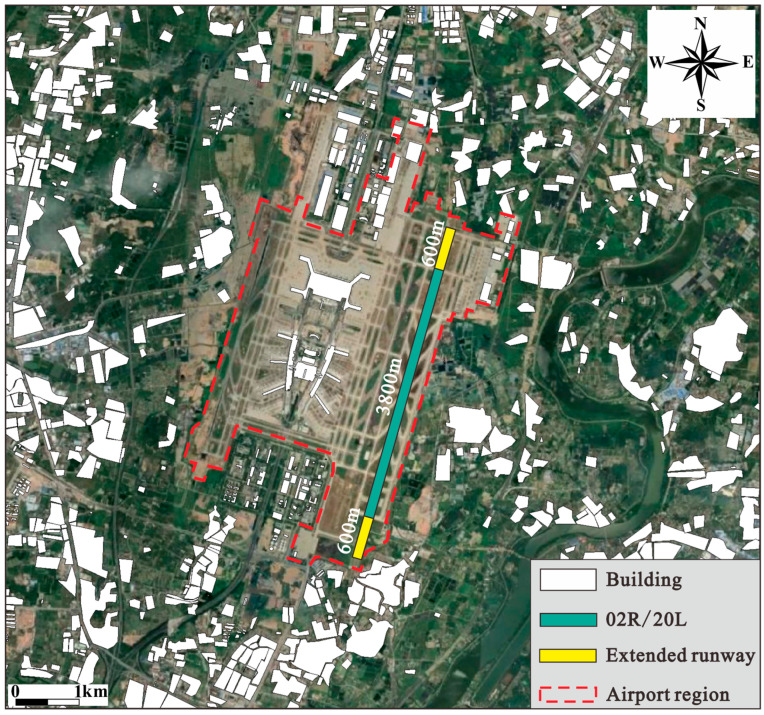
The proposed modification of the operating runway 02R/20L.

As noticed in the aforementioned results, the main areas under high noise levels are often close to both ends of the runway. Hence, it is proposed to achieve better acoustic performance of the airport by regulating the operating scheme of the runways. In this study, the runway 20L/02R of BIA (for landing only) was taken as the objective to investigate aircraft noise under the modified operating schemes in summer and winter, as shown in Figure 9. Specifically, the modifications are aimed at making the actual operating runway closer to the airport itself so that the affected area under the propagation of aircraft noise at landing can be reduced. The proposed operating schemes of runway 20L/02R in summer and winter are as follows.

**Figure 10:**
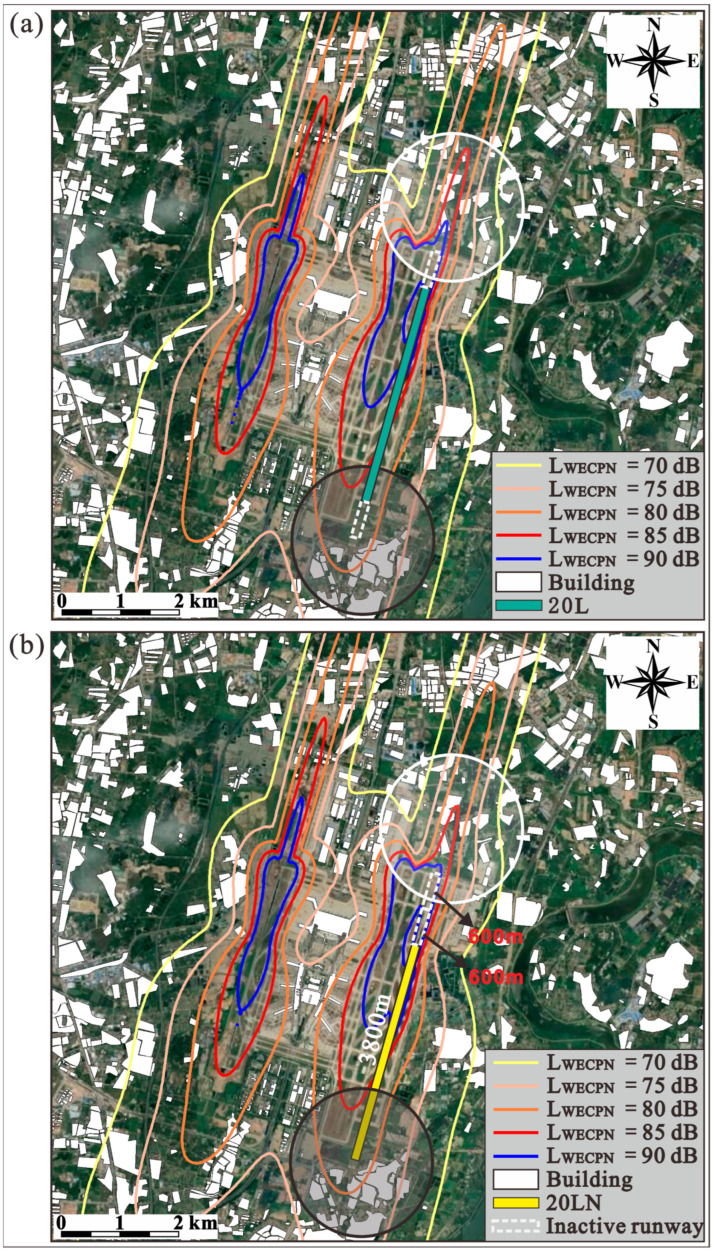
Noise contours (**a**) before and (**b**) after runway extension during summer.

**Figure 11:**
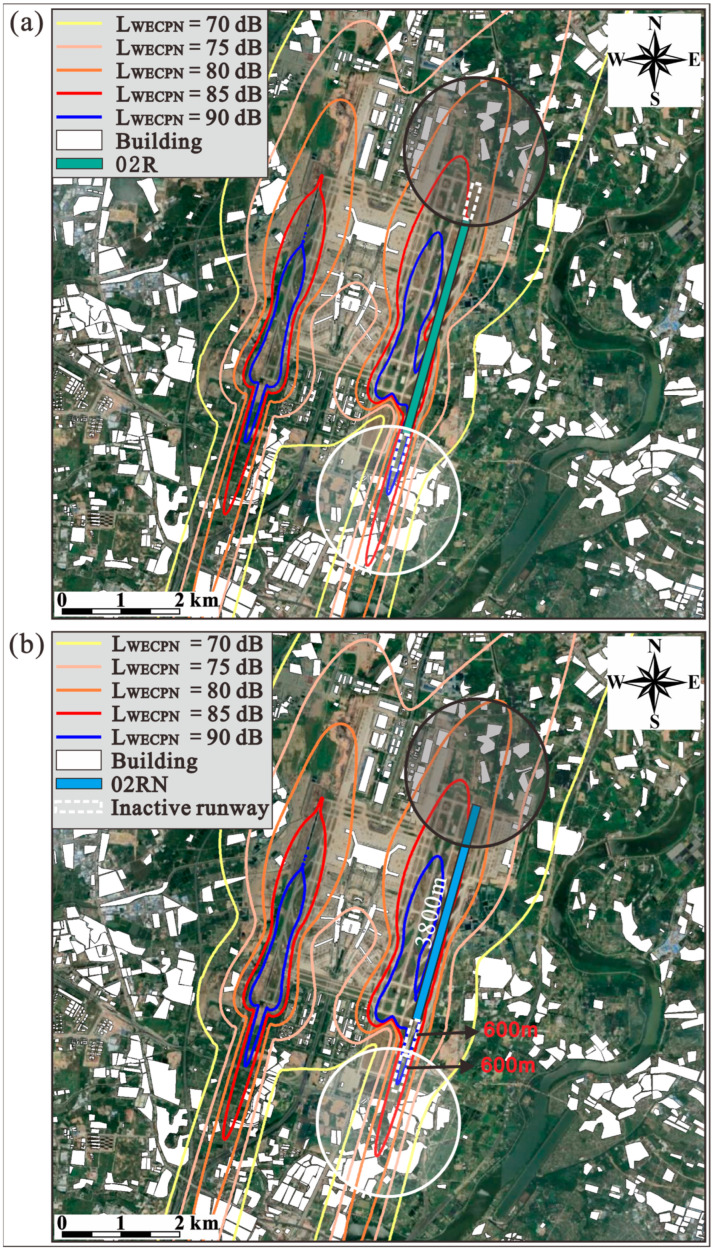
Noise contours (**a**) before and (**b**) after runway extension during winter.

1. It is assumed that runway 20L/02R (3800 m) can be extended in both south and north directions by 600 m, by which the extended runway is still located within the airport region, as shown in Figure 9;
2. **Summer:** The wind is mainly blowing from south to north direction, BIA only uses the proposed new runway 20LN as shown in Figure 10. The white and black circles are the end of the landing and non-landing zones of the runway, respectively;
3. **Winter:** The wind is mainly blowing from north to south direction, BIA is assumed to use the new runway 02RN as shown in Figure 11. The white and black circles are the end of the landing and non-landing zones of the runway, respectively.

**Figure 12:**
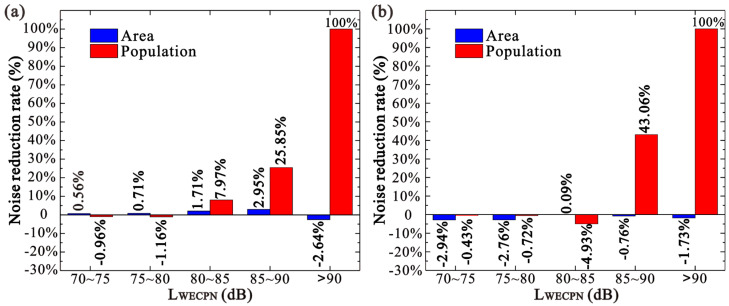
Noise reduction rates by extending the runway during (**a**) summer and (**b**) winter.

It should be noted that the lengths of these modified operating runways are still 3800 m, meaning the operating runways 20L/02R are overall moved 600 m to the south and north directions during summer and winter, respectively. As shown in Figure 10 and Figure 11, it is obvious that by moving the runways in summer and winter, the areas covered by the noise profiles of higher noise levels such as $L_{\mathrm{WECPN}}$ = 85 dB and 90 dB are reduced significantly at the end of the landing zone (white circles). Consequently, the population exposed to high noise levels decreases significantly, as tabulated in Table 5. For instance, the exposed population in the area with $L_{\mathrm{WECPN}}$ > 85 dB decreases by 32.17% and 49.22% during summer and winter, respectively. In addition, no more people are exposed to noise levels higher than 90 dB. It is worth mentioning that although the non-landing zones (black circles) of the new runways (20LN/02RN) are closer to the residential buildings, the simulation results show that the noise profiles of the non-landing zones do not change significantly. This is because the taxiing distance required for an aircraft to land is always shorter than the length of the runway [32], so no aircraft is taxiing at the end of the non-landing zone. Figure 12 clearly indicates that the modification of the operating runway is more effective at alleviating unfavorable effects on the population that suffers under a high noise level (i.e., $L_{\mathrm{WECPN}}$ > 85dB). Hence, the application of such an approach would be superior when the population is dense at the near end of the landing zone.

### 2.5. Land Use Planning

**Figure 13:**
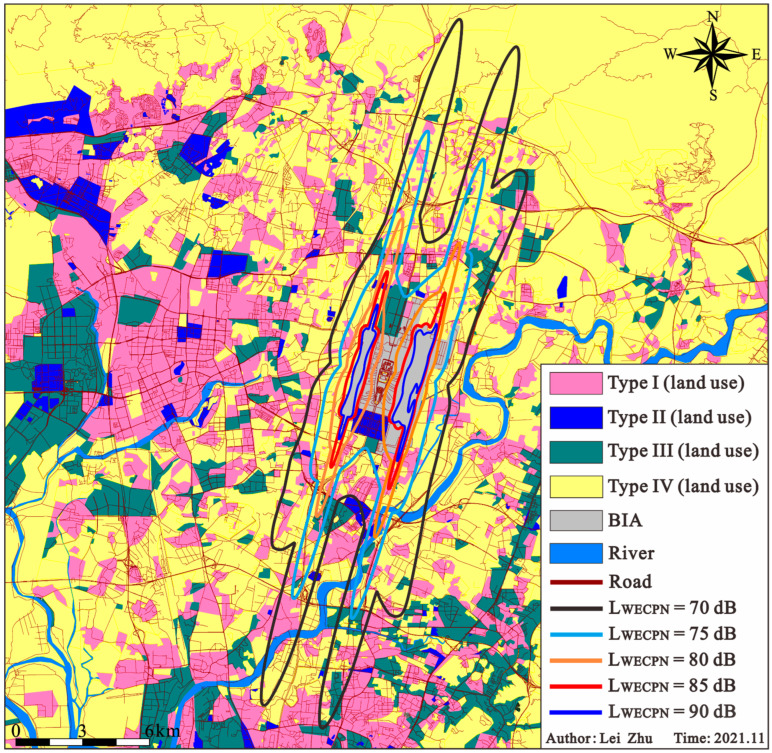
Land use map around BIA.

To minimize the effects of aircraft noise on the surrounding areas of an airport, good land use planning is another active approach. Reasonable planning of land use can not only control aircraft noise pollution effectively, but also allows the harmonious development of the airport and society simultaneously. As shown in Figure 13, the existing land use map around BIA is plotted based on the categorization from Table A2. Based on the practices of the Federal Aviation Administration [33] and published methodology [34,35], the land use planning around BIA is determined as shown in Table 6. In this table, “Y” and “N” indicate that buildings are allowed and not allowed to be built, respectively. The numbers 20/25/30/35 indicate that the existing buildings in the area shall conduct noise reduction measures to make sure the differences between the outdoor and indoor noise levels shall reach at least 20/25/30/35dB, respectively. “N (20/25/30)” indicates that it is not allowed to construct new buildings. If the construction of a new building is necessary, the differences between the outdoor and indoor noise levels shall reach at least 20/25/30 dB for the new buildings. Furthermore, “M” in Table 6 indicates that the residents living in these areas should be relocated to avoid harmful effects from the high aircraft noise. With such analysis, relevant departments can properly plan the land use around BIA by referring to Figure 13 and Table 6.

### 2.6. Installation of Sound Insulation Windows

Aside from the aforementioned active approaches, a passive approach such as installing sound insulation windows could be another viable way, according to Section 2.5. In our previous studies [36,37], sound insulation windows have been successfully developed and employed to mitigate outdoor noise such as traffic noise in a residential building. Furthermore, in an effort to reduce the impact of outdoor aircraft noise, researchers have also tried to take similar sound insulation measures in airports in Spain [38]. In the present study, efforts have been also made to guide the installation of sound insulation windows near BIA according to the noise map obtained from the CadnaA analysis.

**Figure 14:**
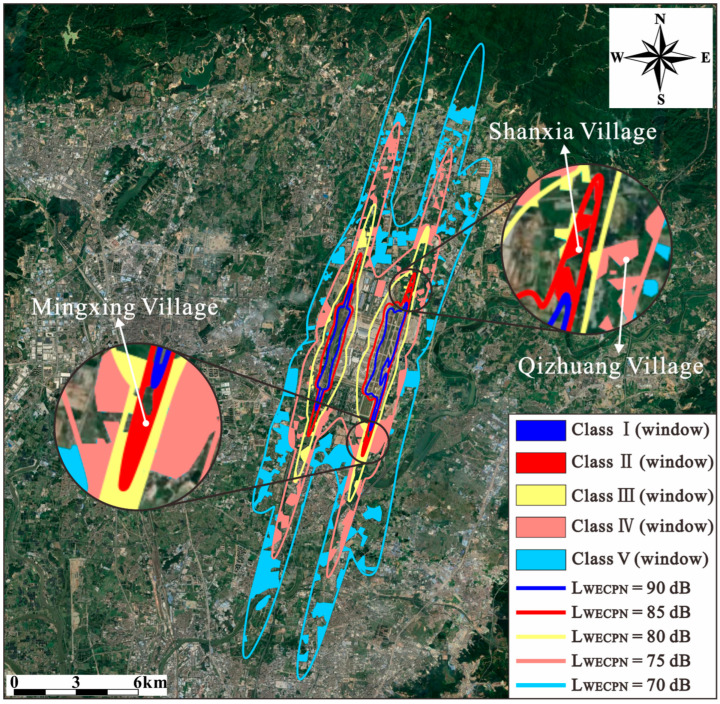
Areas around BIA where different grades of sound insulation windows are required.

Table 7 tabulates the grades of sound insulation windows in terms of their weighted sound reduction index $R_{w}$ and their potential application areas based on $L_{\mathrm{WECPN}}$. The standards of grades are according to the regulations of “Windows for Sound Insulation” (HJ/T17-1996) [39] and “Code for Design of Sound Insulation of Civil Buildings” (GB 50,118-2010) [40]. It should be noted that the sound insulation windows indicated in Table 7 are supposed to be installed in noise-sensitive buildings such as schools, hospitals, etc. As shown in Figure 14, with the assistance of the noise map, the grades of sound insulation windows to be installed in different areas surrounding BIA can be determined. In particular, the buildings in the areas usually suffering under high noise levels, such as those near both ends of runway 20L/02R, have been enlarged for a better view. As can be seen, for these areas at the ends of runway 20L/02R, better grades of sound insulation windows are necessary to protect people from the annoyance of aircraft noise indoors.

## 3. Conclusions

In this study, six noise reduction measures were proposed to improve the acoustic environment of an international jumbo airport (BIA). CadnaA software was the simulation platform used for the data analysis. The effectiveness of these strategies in reducing the impact of aircraft noise was assessed according to the exposed areas and populations under different noise levels. The results showed that by using low-noise aircraft (A320-232) instead of high-noise aircraft (B737-800), the area and population around BIA under different noise levels can be effectively reduced. Under the noise level $L_{\mathrm{WECPN}}$ > 70 dB, the overall exposed area and population can be decreased by 17.90% and 26.61%, respectively.

Reducing the number of night flights is found to be most significant factor in decreasing the overall noise level of BIA. In particular, the implementation of a curfew could reduce the noise-exposed area and population by 39.03% and 43.43%, respectively. In terms of the geographical conditions near BIA, change of flight procedures is an effective way to reduce the population under the noise level ranging from 70 dB to 75 dB (18.45% and 10.94%) during summer and winter, respectively. Nevertheless, this approach will not help in reducing the noise-exposed area. Based on the unique climate characteristics of BIA, the modified operating runway 02R/20L (extended at both ends) could greatly reduce the population suffering under a high noise level $L_{\mathrm{WECPN}}$ > 85 dB (32.17% and 49.22% reduction during summer and winter, respectively) without exceeding the airport region.

In addition, this study has also made an effort to construct a land use map around BIA and develop a new land use plan based on different land types. With such a plan, more people can be protected from being affected by aircraft noise. Furthermore, with the assistance of the noise map, the grades of sound insulation windows to be installed in different areas surrounding BIA can be determined according to the national standards. These results could be of great help for relevant departments to develop policies for existing or new buildings to adopt proper sound insulation windows to ensure healthy acoustic conditions in indoor environments. It is believed that the outcomes of the current study can not only be used as references to reduce the aircraft noise level of BIA, but also provide valuable guidelines for other large-scale airports to mitigate aircraft noise.

## Figures and Tables

**Table 1:** Information on the main aircraft types at BIA [30].

Type	Passenger Capacity (Person)	Price (USD)
B737-800	162	106 million
A320-211	150	98 million
A320-232	150	98 million
A321-232	185	115 million
A330-343	277	256 million
B787-8R	242	248 million

**Table 2:** Noise reduction effects due to aircraft type optimization during summer.

		$L_{\mathrm{WECPN}}$ > 70 dB	$L_{\mathrm{WECPN}}$ > 75 dB	$L_{\mathrm{WECPN}}$ > 80 dB	$L_{\mathrm{WECPN}}$ > 85 dB	$L_{\mathrm{WECPN}}$ > 90 dB
Area (${\mathrm{km}}^{2}$)	Before	83.885	38.813	16.925	7.165	2.960
	After	68.872	32.553	14.307	6.022	2.526
	Percentage	17.90%	16.13%	15.47%	15.95%	14.66%
Population (person)	Before	194,842	75,514	13,414	1831	155
	After	142,995	57,302	9016	1785	146
	Percentage	26.61%	24.12%	32.79%	2.51%	5.81%

**Table 3:** Noise reduction effects by reducing the number of night flights in two stages during summer. The exposed area and population before implementation of any noise reduction strategy (see Table 2) are not shown for brevity.

			$L_{\mathrm{WECPN}}$ > 70 dB	$L_{\mathrm{WECPN}}$ > 75 dB	$L_{\mathrm{WECPN}}$ > 80 dB	$L_{\mathrm{WECPN}}$ > 85 dB	$L_{\mathrm{WECPN}}$ > 90 dB
Practice 1	Area (${\mathrm{km}}^{2}$)	After	68.808	31.328	13.683	5.675	2.300
		Percentage	17.97%	19.29%	19.16%	20.80%	22.30%
	Population (person)	After	154,814	54,225	5682	749	35
		Percentage	20.54%	28.19%	57.64%	59.09%	77.42%
Practice 2	Area (${\mathrm{km}}^{2}$)	After	51.148	23.225	10.040	4.037	1.573
		Percentage	39.03%	40.16%	40.68%	43.66%	46.86%
	Population (person)	After	110,219	30,283	2793	312	0
		Percentage	43.43%	59.90%	79.18%	82.96%	100%

**Table 4:** Noise reduction effects by optimizing the flight procedure during summer and winter.

			$L_{\mathrm{WECPN}}$ > 70 dB	$L_{\mathrm{WECPN}}$ > 75 dB	$L_{\mathrm{WECPN}}$ > 80 dB	$L_{\mathrm{WECPN}}$ > 85 dB	$L_{\mathrm{WECPN}}$ > 90 dB
Summer	Area (${\mathrm{km}}^{2}$)	Before	83.885	38.813	16.925	7.165	2.960
		After	83.535	38.505	16.942	7.164	2.959
	Population (person)	Percentage	0.42%	0.79%	−0.10%	0.01%	0.03%
		Before	194,842	75,514	13,414	1831	155
		After	158,803	65,929	14,205	1831	155
		Percentage	18.50%	12.69%	−5.90%	0%	0%
Winter	Area (${\mathrm{km}}^{2}$)	Before	78.386	36.249	15.790	6.536	2.594
		After	79.913	36.745	15.957	6.582	2.610
	Population (person)	Percentage	−1.95%	−1.37%	−1.06%	−0.70%	−0.62%
		Before	223,261	76,473	22,260	4033	436
		After	198,830	75,579	22,288	4033	436
		Percentage	10.94%	1.17%	−0.13%	0%	0%

**Table 5:** Noise reduction effects by extending the runway during summer and winter.

			$L_{\mathrm{WECPN}}$ > 70 dB	$L_{\mathrm{WECPN}}$ > 75 dB	$L_{\mathrm{WECPN}}$ > 80 dB	$L_{\mathrm{WECPN}}$ > 85 dB	$L_{\mathrm{WECPN}}$ > 90 dB
Summer	Area (${\mathrm{km}}^{2}$)	Before	83.885	38.813	16.925	7.165	2.960
		After	83.263	38.445	16.712	7.119	3.038
		Percentage	0.74%	0.95%	1.26%	0.64%	−2.64%
	Population (person)	Before	194,842	75,514	13,414	1831	155
		After	195,191	74,721	11,901	1242	0
		Percentage	−0.18%	1.05%	11.28%	32.17%	100%
Winter	Area (${\mathrm{km}}^{2}$)	Before	78.386	36.249	15.790	6.536	2.594
		After	80.256	36.881	15.857	6.611	2.639
		Percentage	−2.39%	−1.74%	−0.42%	−1.15%	−1.73%
	Population (person)	Before	223,261	76,473	22,260	4033	436
		After	223,194	75,778	21,173	2048	0
		Percentage	0.03%	0.91%	4.88%	49.22%	100%

**Table 6:** Land use planning under different noise levels ($L_{\mathrm{WECPN}}$) around BIA. “Existing” indicates the existing buildings, “new” indicates the new buildings.

Land Type	Cases	<70 dB	70∼75 dB	75∼80 dB	80∼85 dB	85∼90 dB	>90 dB
I	Existing	Y	20	25	30	M	M
	New	Y	N (20)	N	N	N	N
II	Existing	Y	Y	25	30	35	M
	New	Y	Y	N (25)	N (30)	N	N
III	Existing	Y	Y	Y	25	30	35
	New	Y	Y	Y	N (25)	N (30)	N
IV	New	Y	Y	Y	Y	Y	Y

**Table 7:** Selection of the sound insulation window for sensitive buildings under different aircraft noise levels [39,40]. $R_{w}$ is the weighted sound reduction index.

Noise Level (dB)	Window Grade	$R_{w}$ (dB)
$L_{\mathrm{WECPN}}$ > 90	I	$R_{w}$ ≥ 45
85 < $L_{\mathrm{WECPN}}$ < 90	II	40 ≤ $R_{w}$ <45
80 < $L_{\mathrm{WECPN}}$ < 85	III	35 ≤ $R_{w}$ <40
75 < $L_{\mathrm{WECPN}}$ < 80	IV	30 ≤ $R_{w}$ <35
70 < $L_{\mathrm{WECPN}}$ < 75	V	25 ≤ $R_{w}$ <30

## Data Availability

Data is contained within the article.

## Nomenclature

Symbol	
$L_{\mathrm{WECPN}}$	Weighted equivalent continuous perceived noise level
R	The distance between the measurement point and the aircraft track
$L_{\mathrm{EPN}}$	Effective perceived noise level
${\overset{ˉ}{L}}_{\mathrm{EPN}\;}$	Energy average of effective perceived noise level
$N_{1}$, $N_{2}$, $N_{3}$	The number of aircrafts during day (7:00∼19:00), evening (19:00∼22:00) and night (22:00∼7:00)
${R\;}_{w}$	Weighted sound reduction index
$L_{\mathrm{WECPN}(l)}$	Noise limit of weighted equivalent continuous perceived noise level
$L_{A(l\;)}$	Noise limit of A-weighted maximum sound pressure level
Abbreviations	
BIA	Guangzhou Baiyun International Airport
NPD	Noise–Power–Distance data
ANP	Aircraft noise and performance
EEC	Eurocontrol Experimental Centre

## Appendix A

**Table A1:** Aircraft model at BIA [29].

Type	Model
B737-800	B737-800
A320-211	A320-231, A320-212, A320-214, A320-215, A320-216, A320-251N, A320-252N
A320-232	A320-231, A320-232, A320-233, A320-271N
A321-232	A21N, A321
A330-343	A330-243, A330-341, A330-342, A330-343
B787-8R	B787-9, B787-8

**Table A2:** Noise limits and sensitivity of different land types around the airport [41]. $L_{\mathrm{WECPN}(l)}$ is the noise limit of weighted equivalent continuous perceived noise level. $L_{A(l)}$ is the noise limit of A-weighted maximum sound pressure level.

Land Type	Coverage	Sensitivity	Noise Limit	
I	Residential, school, hospital, etc.	Sensitive	$L_{\mathrm{WECPN}(l)}$ = 70 dB	$L_{A(l)}$ = 90 dBA
II	Administrative, commercial, etc.	Quite sensitive	$L_{\mathrm{WECPN}(l)}$ = 75 dB	
III	Industry, warehousing, entertainment, park, plaza, etc.	Less sensitive	$L_{\mathrm{WECPN}(l)}$ = 80 dB	-
IV	Transportation, public facilities, mining, agriculture, waters, etc.	Not sensitive	-	
